# Determining the frequency of burn wound dressing for clinically competent nursing students: establishing standards based on learning curves

**DOI:** 10.1186/s12909-023-04673-8

**Published:** 2023-09-18

**Authors:** Batoul Alizadeh-Taghiabad, Seyyed Reza Mazloum, Kheizaran Miri, Mohammad Namazinia

**Affiliations:** 1 Department of Nursing, Neyshabur University of Medical Sciences, Neyshabur, Iran; 2 Nursing and Midwifery Care Research Center, Mashhad University of Medical Sciences, Mashhad, Iran; 3 Department of Medical, Surgical Nursing, School of Nursing and Midwifery, Mashhad University of Medical Sciences, Mashhad, Iran; 4 Department of Nursing, School of Nursing and Midwifery, Torbat Heydariyeh University of Medical Sciences, Torbat Heydariyeh, Iran

**Keywords:** Burn wound dressing, Nursing student, Clinical competency, Learning curves, Standards

## Abstract

**Background:**

The primary objective of clinical practice in nursing education is to achieve mastery of clinical skills through repetitive practice. Therefore, there exists a correlation between the frequency of skill demonstration and clinical competency. This study aimed to address the following question: How many times should a nursing student perform burn wound dressing to attain clinical competency?

**Methods:**

This time series study was conducted on 41 junior nursing students who were selected through a census sampling method at Neyshabur School of Nursing from spring 2015 to summer 2021. The data collection tool was a researcher-made competency evaluation checklist (CEV). The competency score of each student for each skill demonstraion episode was evaluated using the Competency Evaluation Checklist (CEV) and recorded on the learning curve until a plateau was reached. SPSS16 and repeated measures analysis of variance (ANOVA) were used.

**Results:**

The mean competency score of burn wound dressing was 67.5 ± 11.0 (out of 100) during the first attempt, which increased to 95.9 ± 4.3 by the ninth attempt. The learning curve for the burn wound dressing skill reached a plateau after five attempts. Additionally, by the fifth attempt of dressing the burn wound, all students could accurately identify the depth, extent, and severity of the wound.

**Conclusions:**

Establishing a standardized frequency for clinical skill performance and ensuring ample practice opportunities can have significant benefits in nursing education systems. These benefits include long-lasting learning, reduced costs, and improved effectiveness. As a result, nurse managers and lecturers should consider the resources available in their educational systems and strive to provide students with adequate opportunities and a supportive environment to practice their skills.

## Introduction

Nursing encompasses both theoretical and practical knowledge, making it a practical science [1, 2]. Nursing education, therefore, should include both theoretical and clinical components. Clinical education is a dynamic and unique process that offers students the opportunity to apply theoretical knowledge to real-world settings [3]. Clinical education accounts for more than 50% of nursing education program, with approximately 1800 h (36 credits) dedicated to internships out of a total of 2500 h (130 credits) in the curriculum [4]. It is widely recognized among educational practitioners that clinical education serves as the foundation and essence of nursing education [5]. The ultimate goal of nursing education programs is to develop clinical competence, which refers to the ability to perform tasks in accordance with expected standards of the nursing profession [6, 7].

Attaining clinical competence is crucial for nurses to possess the necessary authority and capability to effectively perform nursing activities [8, 9]. Therefore, nursing education should prioritize the development of a high level of competence in nursing care among students. Psychomotor skills, which are an integral part of clinical education, play a significant role in clinical competence [3, 10]. Without mastering these skills, nursing graduates may lack the necessary clinical competence, resulting in deficiencies in patient care. However, studies indicated that nursing students, graduates, instructors, and nurse managers often deemed the proficiency level of nursing graduates insufficient. Mazloum (2011) highlighted that some nursing students did not have the opportunity to practice certain clinical skills during training [11]. Additionally, their findings revealed that during the training period, 70% of senior nursing students performed burn dressings less than twice [12].

Global statistics indicate that approximately six million individuals seek burn treatment each year, with the majority receiving care in outpatient clinics [13]. However, research has shown that a significant percentage of nursing students lack adequate knowledge regarding initial care for burn victims, with only 22.4% demonstrating sufficient understanding [13–15]. Another study revealed that the majority of nurses exhibited poor knowledge and incompetence in burn care practices [16]. However, a separate study demonstrated that the utilization of simulation methods such as high-fidelity human patient simulators could effectively enhance nursing students’ knowledge and skills in planning care for burn patients [17]. These findings highlight the need for improved teaching strategies and training programs aimed at enhancing nursing students’ knowledge and proficiency in burn care management [18].

The frequency of skill demonstrations directly correlates with the enhancement of clinical competence in nurses [19, 20]. Therefore, it is crucial to ensure that skills are practiced frequently during clinical training. To determine the standard number of times a skill should be demonstrated, the level of mastery achieved with each demonstration can be assessed and recorded, which is known as the learning curve. Learning curve theory is based on the principle that the time required to perform an activity decreases with repeated practice. This represents the changes in proficiency that occur as a result of performing a skill multiple times. This concept has been applied in medical education across various fields such as in gastrointestinal, cardiac, and orthopedic surgeries [21–25]. Similarly, nursing students have been involved in studies examining general and specialized skills [11, 26–28].

The learning curve is represented graphically in a coordinate system [29], where the horizontal axis represents the frequency of skill demosntration and the vertical axis represents the level of mastery [25]. This curve allows the evaluation of students’ learning rates. The concept of the learning curve aligns with Banner’s novic to expert theory, which suggests that repeated practice of a skill leads to a gradual increase in overall clinical proficiency [30]. By plotting a learning curve, a standard for the frequency of performing clinical skills can be established. However, in the review of reliable nursing sources, including books on nursing principles and techniques, no specific information was found regarding the number of times required to master these techniques. This lack of information may be attributed to various factors such as learners’ conditions, educational environments, and other contextual factors, making it challenging to establish an international standard in this field.

Therefore, it is necessary to develop local standards for this domain. Developing educational standards promotes evidence-based education and enhances the quality and efficiency of education. Standardizing the clinical nursing skills training program offers several benefits, including optimizing the utilization of educational resources, documenting the scheduling of the nursing training program, designing a clinical skills record book, modifying the training program, and ultimately enhancing the clinical competence of nursing graduates [31]. This study aimed to determine the appropriate educational standard for burn dressing skills by smoothing the learning curve and achieving an average proficiency of 75%.

## Methods

### Study design

The current time series study was conducted at the burn department of Imam Reza hospital in Mashhad, Iran, from spring 2015 to summer 2021. As part of their practical training, these students underwent a five-day apprenticeship in the burn department of Imam Reza hospital during the summer.

### Participants

The study population consisted of 41 junior undergraduate nursing students from Neyshabur Faculty of Medical Sciences. The inclusion criteria were nursing students at Neyshabur Faculty of Nursing and Midwifery, who received theoretical and practical training in the principles and techniques of nursing, and received theoretical training in internal surgery skills, including burn dressings. The exclusion criteria were students who had not performed the desired skill in a clinical setting and those who were not mentally or psychologically prepared to perform the skill due to factors such as acute stress.

### Outcomes

A demographic data questionnaire and Competency Evaluation Checklist were used to collect data. The demographic data questionnaire had six questions about age, average grades, interest in nursing, work experience in the hospital, and work experience in the burn department. This questionnaire was administered through interviews with the participants. A researcher-conducted checklist called the Competency Evaluation Checklist (CEV) was used to assess proficiency in burn dressing skills. The content validity of both the demographic information questionnaire and the CEV checklist was established by consulting the latest books and articles on the research topic. These forms were then reviewed and revised based on feedback from 10 faculty members of the nursing faculty. The CEV checklist was further validated by computing the content validity index (CVI = 0.9) and assessing the intrarater reliability coefficients (r = 0.91). The checklist comprised 42 items, which were categorized as follows: three items for diagnosing and conducting preliminary examinations of burn wounds, 34 items for washing and changing dressings, and five items for report writing. These items were used to assess various aspects of clinical skills. Each item was evaluated using three options: “Able to perform independently” (score 3), “Able to perform with assistance” (score 2), and “Unable to perform” (score 0). The overall competency score for each skill ranged from a minimum of zero to a maximum of 100.

### Sample size

The sample size was determined based on the pilot study and the mean estimation formula for the “burn dressing skill.” This calculation was performed with a confidence interval of 95% for a minimum of 10 individuals with burn dressing skill. Since the measurement of proficiency did not continue after reaching 100% proficiency or the smooth line of the learning curve, it was necessary to include 41 people in the study to ensure a sufficient number of research units at the end. Additionally, three to four samples were replaced each time. Towards the end of the study, some individuals may have dropped out because they performed skills without registration. As a result, a proficiency test was conducted with the remaining 10 participants.

### Data collection

The level of clinical competency was assessed multiple times throughout the study to evaluate the students’ clinical skills and their impact. This evaluation process continued until the learning curve became smooth, with less than a five-percent change or 100% competency.

On the first day of the apprenticeship, the instructor introduced the department’s appearance, personnel, rules, and routine to the students. Prior to starting this apprenticeship course, the students had already learned the theoretical and practical aspects of dressing and bandaging in the Principles and Techniques course. They also practiced simple wound dressing in internal-surgical departments. Although they had theoretical knowledge of different types of burn wounds, they had not yet observed such wounds in practice or performed dressing on them.

To reinforce and recall theoretical information, oral training was conducted using the Competency Evaluation Checklist (CEV) to guide the steps and procedures for performing burn wound dressing. These steps included washing hands, gathering the necessary equipment, wearing masks and scrubs, identifying the wound, cleaning and dressing it, providing patient education, and documenting the procedure.

After explaining the study objectives and obtaining written informed consent, we collected the demographic characteristics of the students by administering a relevant questionnaire. Subsequently, students observed at least two dressing processes conducted by ward nurses. While the students had previous familiarity with simple dressings and bandages from previous semesters, they lacked experience dressing burn wounds.

Under the supervision of the instructor, the students independently dressed burn wounds. During this process, the researcher completed the Competency Evaluation Checklist (CEV). Data were collected by the first author of the study (BAT).

The competency score for each student’s skill demonstration was evaluated using the Competency Evaluation Checklist (CEV) and recorded on a learning curve until a plateau was reached. The evaluation was considered complete when the learning curve reached a plateau (with a change of less than 5% between repetitions of skill demonstration) or when the student achieved 100% competency [5].

### Statistical analysis

The data were analyzed using SPSS16. Descriptive statistics, such as the frequency distribution, mean, and standard deviation, were used to summarize and report the variables. Inferential statistics, including repeated measures analysis of variance (ANOVA), were used to compare the mean competency scores of procedural skills of burn wound dressing in the burn ward at different times. Additionally, correlation coefficient tests and linear regression model were employed.

## Results

Among the 41 nursing students who participated in the study, 87.8% were female. The mean age of the students was 22 ± 0.97 years, ranging from 20 to 24 years. The students’ grade point average (GPA) in previous semesters ranged from 12.80 to 19 out of 20, with an average of 16.82 ± 1.25. Furthermore, 58% of the students expressed a high level of interest in nursing, while 12.2% indicated a low level of interest in their field of study. The findings revealed that 22% (n = 9) of the students had previous experience working in a hospital, but none of them had specifically worked in the burn department (Table 1).

**Table 1:** Demographic characteristics of the nursing students participating in the research

Variable		N
Gender		
	Female	36 (87.8%)
	Male	5 (12.2%)
Age (years)		
	Mean ± SD	22 ± 0.97
	Range (Mini- Maxi)	(20–24)
Grade point average (GPA)		
	Mean ± SD	16.82 ± 1.25
	Range (Mini- Max)	(12.8–19)
Interested in Nursing		
	Very moderate	58.0% 29.8%
	Little	12.2%
History Of Student Work In The Hospital		
	Yes	9 (22%)
	No	32 (78%)
Worked In The Burn Department		
	Yes	0
	No	100%

During summer apprenticeship, junior undergraduate nursing students in the burn department performed burn dressing clinical skills for 205 patients. The students’ skill demonstrations ranged from a minimum of two times to a maximum of nine times. The mean competency score for burn dressing skill was 67.6 ± 11.0 on the first attempt, which increased to 95.0 ± 3.4 by the fifth attempt (Table 2). Furthermore, 82.5% of the students were able to accurately determine the extent of the burn independently by the third attempt, and 70.7% were able to determine the depth of the burn by the fourth attempt (Table 3). The repeated measures ANOVA test results indicated significant differences in the mean competency scores across different attempts. Some students reached the cut-off points by performing a skill three times, with changes of less than 5%, while others performed the skill up to ten times. The average standards were considered in these cases (Table 2).

**Table 2:** The average level of proficiency in performing the clinical skill of changing the burn dressing according to the number of times it is performed in the studied nursing students

Skill Performance Time	Number Of Persons	Proficiency Level	The Percentage Of Changes In Proficiency Compared To The Previous Time
		M ± SD	
1	41	67.6 ± 11.0	-
2	41	80.3 ± 9.3	19
3	40	87.3 ± 5.6	8
4	33	91.4 ± 3.9	4
5	17	95.0 ± 3.4	3
6	14	94.1 ± 2.2	0
7	11	96.8 ± 2.7	2
8	5	95.4 ± 1.2	1
9	3	95.9 ± 4.3	0
The Result Of Analysis Of Variance With Repeated Measurement		P = 0.000	

**Table 3:** Frequency distribution of studied nursing students according to the degree of proficiency in the diagnosis of burn wounds according to the number of times it is performed

Frequency of performing clinical skills	NO	Proficiency in burn wound diagnosis		
		depth	extent	Degree
1	41	7 (17.1)	9(22)	6(18.8)
2	41	16(32)	15(36.6)	16(39)
3	40	30(75)	33(82.5)	20(62.5)
4	33	29(70.7)	32(97)	20(62.5)
5	17	17(100)	17(100)	17(100)
6	15	14(100)	14(100)	14(100)
7	15	10(90)	10(100)	10(100)
8	10	5(100)	5(100)	5(100)
9	10	3(100)	3(100)	3(100)

**Fig. 1:**
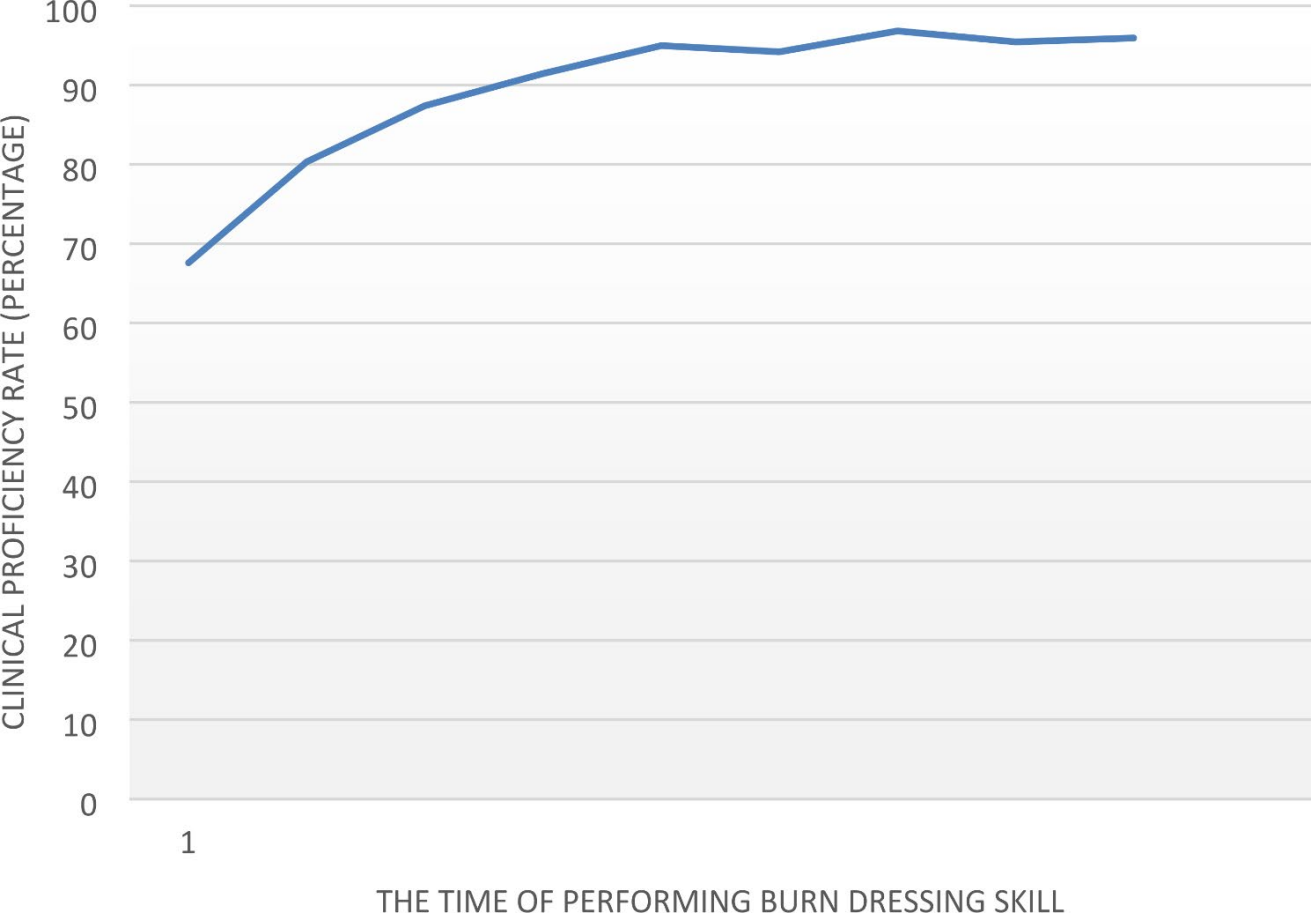
The learning curve of the studied nursing students’ proficiency in burn dressing skills

On average, the students achieved competence in performing the desired burn dressing skill after five attempts. The learning curve reached a plateau, with changes of less than 5% between the fourth and fifth attempts (Table 2). Figure 1 illustrates the learning curve of students’ competence in successive attempts of the burn dressing clinical skill. It shows an upward trend in skill demonstration from the second to the fifth attempt, with a gradual improvement (Fig. 1). By the fifth attempt, the change in skill demonstration was less than 5% compared to the fourth attempt. The learning curve plateaued after five attempts, which is considered the standard frequency for clinical skill demonstration. The mean competency score in the fifth attempt was 94.97 ± 3.36.

In the current study, there was no significant correlation between the percentage of increase in competency in the first attempt compared to the last attempt and variables such as age, GPA of past semesters, level of interest in nursing, and history of clinical experience. However, the Pearson correlation test revealed a linear correlation between the grade of the theoretical course on burns and the percentage of “increase in competency in the first attempt compared to the last attempt” (p = 0.002 and r = 0.8). Additionally, the independent t-test indicated that the mean percentage of “increase in competency in the first attempt compared to the last attempt” in the clinical skill of changing burn dressings was higher in men than in women (p = 0.005).

## Discussion

In this study, the proficiency level of students in performing burn dressing skills was recorded using a standard checklist. The learning curve was then analyzed, and three smoothing indicators were used to determine the average time it took for students to achieve a proficiency level of 75%.

The analysis of the learning curve revealed that competency in burn dressing skills reached a plateau, with less than a five-percent change or 100% competency after five attempts. The study conducted a literature review and found no articles specifically examining the learning curve for identifying and dressing burn wounds among nursing students. However, the study referenced other research that developed standards based on the learning curves for various nursing skills. For instance, one study indicated that competency in measuring blood pressure, pulse rate, and respiration rate could be achieved after five attempts, whereas changing dry dressings and infusing intravenous fluids required six attempts. According to one study, only 22.4% of nursing students demonstrated adequate knowledge in providing initial care for burn victims [13, 14]. Another study highlighted that the majority of nurses exhibited poor knowledge and incompetence in burn care practices. These findings clearly indicate the necessity for enhanced teaching strategies and training programs to improve nursing students’ knowledge and skills in managing burn care [18].

The research estimated that at least five attempts were required during apprenticeship to achieve competency in burn dressing performance. However, 82.5% of the students were able to accurately and independently determine the extent of the burn by the third attempt, and 70.7% could determine the depth of the burn by the fourth attempt. The learning curve analysis of students’ competence level in changing burn dressings revealed an overall upward trend, with a slight decrease observed in the sixth episode. This decrease may be attributed to a decrease in the accuracy and concentration on certain details due to increased competence among learners. It is possible that learners develop a false sense of confidence in their proficiency and experience a decrease in the anxiety and fear associated with skill demonstration. This phenomenon aligns with the concept of the feeling of mastery that often emerges in the early stages of skill acquisition. It is important to note that specialized and complex skills, particularly in critical patient conditions, may require more attempts to achieve competency. The concept of learning curve has been studied in various fields of medical education, including the acquisition of surgical techniques in gastrointestinal, cardiac, and orthopedic surgeries [21–25, 27].

A study conducted in Philadelphia, United States revealed that pediatric critical care medicine fellows required an average of 19–54 attempts to achieve a 90% overall success rate in performing tracheal intubation [32]. In comparison, research indicates that for adult tracheal intubation techniques, which are generally less complex than pediatric ones, paramedic students in Pittsburgh, Pennsylvania achieved a success rate of over 90% with an average of 15 to 25 attempts on live patients in the operating room [23]. However, the learning curve did not plateau even after 30 attempts in pre-hospital, emergency, and intensive care units [33]. It appears that the setting in which a skill is performed also influences the formation of the learning curve. For instance, students tend to quickly gain proficiency in tracheal intubation in the operating room, where patients have already received anesthetics and sedatives. Nurse anesthesia trainees in Thailand required a minimum of 22 attempts to achieve an 80% success rate within the tracheal intubation technique when they had no prior experience in this field [32]. In another study focusing on laparoscopic colorectal surgery, researchers examined two subspecialty surgical and laparoscopic units in the United States and United Kingdom. They found that 50 procedures were sufficient to complete the learning curve for a particular surgery. The study also analyzed the average operative time for the right and left incisions separately as part of the learning curve assessment. The mean operative time for the left incision decreased from 251 min in the first 25 cases to 187 min in the second 25 cases. Similarly, the mean operative time for the right incision decreased from 200 min in the first 25 cases to 147 min in the second 25 cases. The feeling of self-confidence in performing the operation and the reduction in operative time were identified as influential factors. Researchers suggest that it is crucial to precisely define important aspects, such as patient positioning, complex procedures, and the roles of nursing staff and assistant surgeons to ensure that each component plays its role effectively [34].

However, relatively few studies have been conducted on the field of nursing. For example, Mazloum et al. (2011) conducted a study on basic clinical skills in nursing, including vital signs, simple dry dressing, and report writing [31]. Miri et al. (2013) studied the skill of establishing an intravenous line and intramuscular injection [11, 27]. Additionally, Vahedian et al. (2021) conducted a study on the clinical skills of nurses regarding arterial blood sampling in the intensive care unit [27].In a study focusing on health workers, the learning curve demonstrated that practicing clinical family planning skills 21–30 times [10] and child care skills 40 times were sufficient to achieve adequate competency [26].

The initial measurement of proficiency in each burn dressing reflects the students’ basic proficiency level, which is influenced by their learning in theoretical and practical courses on nursing principles and the theoretical course on internal surgery, specifically focusing on the skin and burns [35]. During these courses, students become familiar with the theoretical foundations of the skills and practice them in a clinical skills center under the supervision of an instructor [36]. The numerical value assigned to this basic proficiency level can be associated with the complexity or simplicity of skills. The difficulty or simplicity of a skill is determined by factors such as its nature, type, and number of steps involved in its execution. In the current study, since the students had previously performed regular dressings and were acquainted with the theoretical aspects, their basic proficiency level (first attempt) for burn dressing skill was reported to be 67.6 ± 11.0.

In the present study, there was a relationship between the grade obtained in the theoretical course on burns and the mean competency rate of students. It was found that students who had lower grades in this course showed a higher increase in competency during the first attempt compared to the last attempt. In other words, these students required more practice to achieve competency.

These findings suggest that theoretical knowledge plays a significant role in influencing clinical competencies. In a study involving health workers, it was observed that they required more practice to achieve competency compared to nursing students, possibly due to the fact that health workers receive less theoretical education [26].

It is important to note that theoretical education in classrooms alone does not determine the performance levels. Performance is influenced by an individual’s level of experience in a clinical setting. Theoretical knowledge serves as the foundation for learning clinical skills, which then expands through clinical experience and practical skill improvement [10, 33].

After five dressing attempts, students generally acquired the necessary skills in this technique. However, it appears that the skill of bandaging and fixing the dressing might require additional practice. Certain body parts such as the head, shoulder, or fingers might require more practice and skill to effectively perform dressing.

Caregiving duration is an important factor in determining the learning curve. However, in the burn department, different areas of the body are involved, and wounds of varying sizes require different amounts of time to perform dressing. Therefore, it is not feasible to consider the duration of the technique used in this study. For example, the extent of burns on the fingers may be considered to be 0.5%, but compared to an area of the wrist with the same extent of burns, dressing the fingers may take more time. Since this study did not separate the learning curve based on dressing different body parts, the recording of dressing time was not applicable in the analysis of the curve.

Another influential factor in the student learning process that should be considered in future studies, is the number of observations of dressing skills performed by trainers. These observations are typically conducted in the presence of students before they begin independently their skills [37, 38].

It is suggested that in the stage of internships, there should be careful planning for the training of more students in the burn department. This plan should consider the factors that influence the persistence and retention of clinical skills such as the type of skill (cognitive or psycho-motor), the complexity of the steps involved, the provision of feedback to students, the method and duration of exercises, the spacing between attempts, and the training setting. Additionally, the personal characteristics of students, including their abilities, memory, and motivation, should be considered.

It is important to note that clinical nursing skills are gradually acquired, and if not continuously and sufficiently practiced, they can weaken over time. In the absence of regular practice, the half-life of many clinical skills is only a few months. Therefore, to ensure the retention and proficiency of clinical skills, it is recommended to provide ongoing opportunities for practice and reinforcement during internships in burn departments.

Although the teaching of psychomotor skills in nursing and medical schools has remained largely unchanged over the past 50 years, there are known issues with the inconsistent teaching, evaluation, and maintenance of critical basic skills. Despite this, the primary focus of educational systems is on improving clinical settings and enhancing patient care [39].

Establishing standards for assessing the clinical competence of students and nurses is crucial in ensuring that students receive high-level clinical skills training. This reduces costs and also leads to the provision of better services. The findings of this study can be applied to the clinical education of nursing students and other healthcare professionals. Additionally, these results can inform educational planning, enabling the development of a supportive and effective clinical educational model and methodology in health-related students.

This study had several limitations that should be acknowledged. One limitation was the difficulty in controlling factors that could influence students’ learning such as motivation, innate talent, and interest in the field of nursing. Another limitation was that while the burn percentage of patients was measured by the students and confirmed by the instructor, the related data were not collected specifically for this study. Furthermore, the study duration was insufficient to achieve a substantial sample size. Additionally, the geographical distance between the clinical training center (Imam Reza hospital in Mashhad) and the theoretical training center (Neyshabur School of Nursing) posed significant challenges for the research team.

## Conclusion

The analysis of the learning curve for students’ competence level in changing burn dressings revealed an overall upward trend in competence across successive skill demonstrations, with a slight decrease observed in the sixth attept. This suggests that more frequent attempts are needed to reach a plateau in skill proficiency based on the learning curve.

To further analyze the data such as by conducting repeated measures ANOVA tests or calculating the learning formula for students, additional dressing attempts are required. However, due to the short duration of apprenticeship in the burn department and the critical importance of learning burn wound care, nursing instructors in the burn department should provide each student with the opportunity to perform at least five episodes of dressing. This will allow students to achieve sufficient proficiency and gain more exposure to different types of burn wounds, therby enabling them to practice care and dressing techniques.

In terms of planning, it is recommended that managers allocate a minimum of ten days of 5-hour apprenticeships for nursing students to develop competency in this particular skill.

## Data Availability

The datasets generated in the current study are available from the corresponding author upon reasonable request.

## List of abbreviations

GPA: Grade Point Average
CEV: competency evaluation checklist
